# Fever Instruction at the Metropolitan Asylums Board Hospitals

**Published:** 1898-01-01

**Authors:** 


### FEVER INSTRUCTION AT THE METROPOLITAN ASYLUMS BOARD HOSPITALS.

A course of instruction in the diagnosis and treatment of fevers will be held at each of the undermentioned hospitals on Tuesdays and Fridays at eleven a.m., commencing Tuesday, January 11th, 1898.

Eastern Hospital, The Grove, Homerton, N.E.—Dr. E. W. Goodall, resident medical superintendent.

North-Western Hospital, Haverstock Hill, N.W.—Dr. W. Gayton, resident medical superintendent.

Western Hospital, Seagrave Road, Fulham, S.W.—Mr. R. M. Bruce, resident medical superintendent.

Brook Hospital, Shooter's Hill, Kent.—Dr. J. MacCombie, resident medical superintendent.

Park Hospital, Hither Green, Lewisham, S.E.—Dr. R. A. Birdwood, resident medical superintendent.

South-Eastern Hospital, Hatfield Street, New Cross, S.E.—Dr. F. M. Turner, resident medical superintendent.

*South-Western Hospital, Landor Road, Stockwell, S.W.—Dr. F. F. Caiger, resident medical superintendent.

Fountain Hospital, Grove Road, Lower Tooting, S.W.—Dr. C. E. Matthews, resident medical superintendent.

* This class being full, a second class has been formed on Mondays and Thursdays, at eleven a.m., commencing Monday, January 10th, 1898.

